# A Single-Institution Retrospective Study of Patients Treated With Laser-Interstitial Thermal Therapy for Radiation Necrosis of the Brain

**DOI:** 10.7759/cureus.19967

**Published:** 2021-11-28

**Authors:** Claire M Lanier, Michael Lecompte, Chase Glenn, Ryan T Hughes, Scott Isom, Wendy Jenkins, Christina K Cramer, Michael Chan, Stephen B Tatter, Adrian W Laxton

**Affiliations:** 1 Department of Radiation Oncology, Wake Forest School of Medicine, Winston-Salem, USA; 2 Department of Neurosurgery, Wake Forest School of Medicine, Winston-Salem, USA

**Keywords:** radiation oncology, brain metastases, stereotactic radiosurgery, radiation necrosis, laser-interstitial thermal therapy

## Abstract

Object

Laser-interstitial thermal therapy (LITT) has been proposed as an alternative treatment to surgery for radiation necrosis (RN) in patients treated with stereotactic radiosurgery (SRS) for brain metastases. The present study sought to retrospectively analyze LITT outcomes in patients with RN from SRS.

Methods

This was a single-institution retrospective study of 30 patients treated from 2011-2018 with pathologically-proven RN after SRS for brain metastases (n=28) or proximally treated extracranial lesions treated with external beam radiotherapy (n=2). Same-day biopsy was performed in all cases. Patients were prospectively followed with Functional Assessment of Cancer Therapy - Brain (FACT-Br), EuroQol-5 Dimension (EQ-5D), Hopkins Verbal Learning Test (HVLT) and clinical history and examination. Adjusted means, standard errors and tests comparing visits to pre-LITT were generated. Kaplan-Meier method was used to estimate time overall survival. Competing risk analysis was used to estimate cumulative incidence of LITT failure.

Results

In our patient population, median time from radiotherapy to LITT was 13.1 months. Median SRS dose and median LITT treatment target volume were 20 Gy (IQR 18-22) and 3.5 cc (IQR 2.2-4.6), respectively. Seventy-seven percent of our patients tapered off steroids within one month. There were only two instances of RN recurrence after LITT, with recurrence defined as recurrence of symptoms after initial improvement. These recurrences occurred at 1.9 and 3.4 months. The three-, six- and nine-month freedom from recurrence rates were 95.7%, 90.9%, and 90.9%. Median survival in our patient population with pathologically confirmed RN treated with LITT was 2.1 years. Regarding the quality of life questionnaires with which some patients were followed as part of different prospective studies, completion rates were 22/30 for FACT-Br, 16/30 for the EQ-5D and 8/30 for HVLT. Quality of life questionnaire results were overall stable from baseline. Mean FACT-Br scores were stable from baseline (17.9, 16.6, 21.4 and 22.8) to three months (18.8, 15.4, 18.4 and 23.4) (p=0.38, 0.53, 0.09 and 0.59). The mean EQ-5D Aggregate score was stable from baseline (7.1) to one month (7.6) (p=0.25). Mean HVLT-R Total Recall was stable from baseline (20.6) to three months (18.4) (p=0.09). There was a statistically significant decrease in mean Karnofsky Performance Scale (KPS) score from baseline (84) to three-month follow-up (75) (p=0.03).

Conclusions

LITT represents a safe and durably effective treatment option for RN in the brain. Results demonstrate a median survival of 2.1 years from LITT with only two recurrences, both within four months of treatment and salvageable. Patient-reported outcomes showed no severe declines after LITT. Quality of life questionnaires demonstrated stable well-being and functionality from baseline. LITT should be considered for definitive treatment of RN, especially in cases where patients have significant side effects from standards medical therapies such as steroids or if steroids are minimally effective.

## Introduction

Approximately 180,000 patients in the US each year are diagnosed with brain metastases [1]. Stereotactic radiosurgery (SRS) has become the standard of treatment for oligometastatic brain metastases [2,3]. Through technological advances, its use has become more widespread than ever before [4]. As the use of SRS continues to grow, patient life expectancies with brain metastases continue to improve [5,6]. Subsequently, practitioners are becoming more aggressive with the size [7] and number [8] of lesions being treated and toxicity risks are becoming more relevant to practitioners who manage brain metastases. One of the well-known side effects of SRS therapy is radiation necrosis (RN). The exact incidence of RN is unknown, however, it is estimated at 5-25% [9]. Traditional treatments for RN have been limited to corticosteroids or surgical resection, though the use of bevacizumab and hyperbaric oxygen have become viable options as well [9-12].

Laser interstitial thermal therapy (LITT) was originally introduced in the early 1980s as a tool for treating brain metastases. It had a number of technological challenges that limited its use. However, with advances in magnetic resonance thermography, real-time thermal imaging and feedback control, its use is experiencing a renaissance [13]. Recent literature has explored its use in the treatment of both brain metastases and RN. A 2018 prospective study exploring the efficacy of LITT in treating brain metastasis progression, whether from recurrence or RN, concluded that LITT could be safely performed while reducing the use of steroids and preserving quality of life, cognition and performance status. In patients with biopsy-proven RN, LITT produced almost 100% lesion control and over 80% survival at six months [14].

Thus, we sought to retrospectively review a larger cohort of patients with biopsy-proven RN to determine the efficacy of LITT in treating RN following prior SRS for brain metastases or proximally treated extracranial lesions treated with external beam radiotherapy. The information in this article was previously presented as an oral abstract presentation at the 2019 ASTRO Annual Meeting on September 16, 2019 [15].

## Materials and methods

Data acquisition

This study was approved by the Institutional Review Board (IRB) at Wake Forest School of Medicine (IRB00043884). Patients were identified for this study through a prospective IRB-approved multi-institutional database for patients who receive LITT. Patients were consented for the individual prospective studies from which this data was compiled. Patients with pathologically-confirmed RN were included in the study. Data on prior radiosurgery parameters, metastasis histology, patient demographic data, date of LITT and imaging results were determined from the electronic medical record. As part of a prospective multi-institutional database, some patients were followed with Functional Assessment of Cancer Therapy - Brain (FACT-Br), EuroQol-5 Dimension (EQ-5D), Hopkins Verbal Learning Test (HVLT). 

Radiosurgical management

Twenty patients were treated with Gamma Knife (Elekta AB, Stockholm, Sweden) prior to development of RN. Six patients were treated on a linear accelerator-based platform. SRS treatment modality was unknown for two patients. Two patients were treated with external beam radiation. Dosing for radiosurgery was generally in accordance with the guidelines from the Radiation Therapy Oncology Group (RTOG) 90-05 study [16].

Surgical management

**Figure 1:**
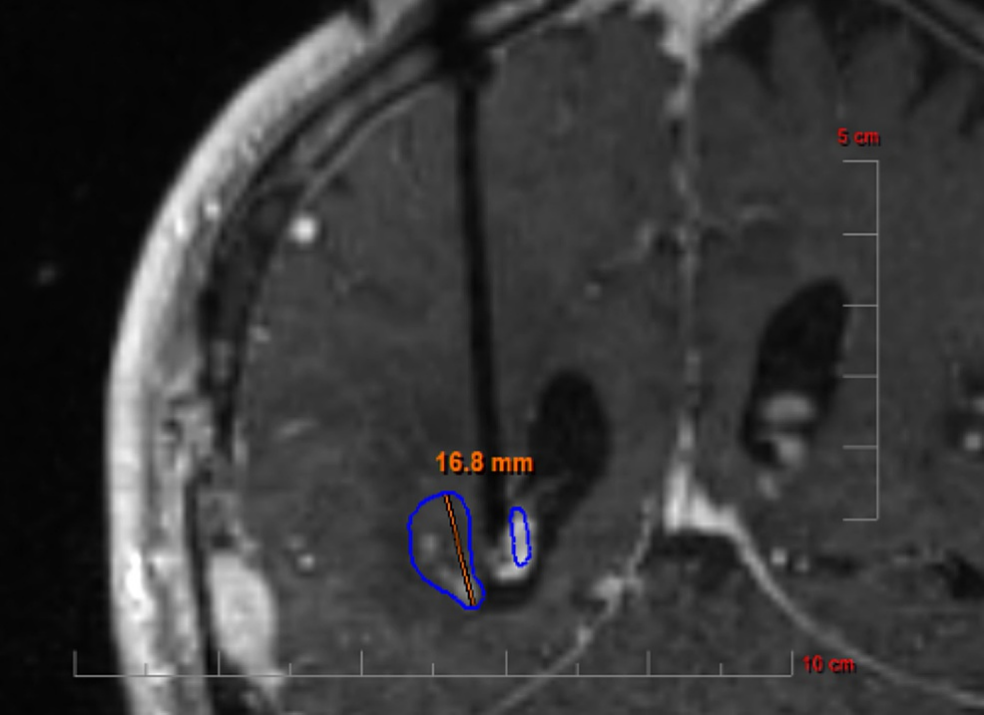
Contrast-enhanced MRI image of a patient undergoing laser-interstitial thermal therapy (LITT) for radiation necrosis. The blue line represents the line inside which biological equivalence of heating to 43˚ C at 10 minutes is delivered.

Pre-operative, same-day, contrast-enhanced MRI was performed to create T1-weighted volumetric images that were used for visualization of the RN lesion and for treatment planning. Same-day biopsy was performed in all cases. LITT was performed using the NeuroBlate system (Monteris Medical, Plymouth, MN, USA) for all cases in this report. In general, a single laser trajectory was used. MRI thermometry was used to predict zones of cell death. The goal of treatment was to treat as much of the enhancing volume of RN as feasible to biological equivalence of 43˚ C at 10 minutes. A sample case of LITT used to treat RN is depicted in Figure 1.

Follow-up and response assessment

Follow-up MRI of the brain was generally performed six to eight weeks after initial LITT procedure and every three months thereafter for the first two years post-procedure. Recurrence of radiation necrosis was defined as return of symptoms requiring intervention after initial improvement following LITT. Recurrence was not defined by imaging findings. LITT's ablation of the necrosis results in an enhancement around the LITT cavity. While that does ultimately decrease overtime, it does not always correlate with symptomatic improvement. In patients who died, cause of death (neurologic vs non-neurologic) was determined from the electronic medical record. Neurologic death was determined based on criteria described by McTyre et al. [17]. In brief, patients who had progressive neurologic decline at time of death (regardless of extracranial disease status) were considered to have died of neurologic death. In addition, patients dying of intercurrent disease but having severe neurologic dysfunction were considered to have neurologic death.

Statistical analysis

To describe the sample descriptive statistics, means and standard deviation or frequency and percentage were used. Not all participants had data for each quality of life measures at each visit as not all patients in this cohort were followed with the same quality of life measures. To account for the missing data, repeated measures linear models were used with visit, treated as a categorical measure, being the only covariate. From these models, adjusted means, standard errors and tests comparing visits to pre-surgery were generated. The Kaplan Meier method was used to estimate overall survival. Both time to event measures originate at the time of LITT and are censored at the date of last follow-up if no event occurred prior to that time. Competing risk analysis with death as a competing risk was used to estimate cumulative incidence of LITT failure. All analyses were done using SAS 9.4 (SAS Institute Inc., Cary, NC, USA).

## Results

Demographics and procedure

Between 2011 and 2018, 30 patients were treated with pathologically proven RN after SRS for brain metastases (n=28) or proximally treated extracranial lesions treated with external beam radiotherapy (n=2). Median time from prior radiotherapy to LITT was 13.1 months. Median SRS dose prior to RN was 20 Gy (IQR 18-22). Primary cancers included lung (43%), non-melanomatous skin/head and neck (20%), breast (17%), melanoma (7%) and other (13%). Median LITT target volume was 3.5 cc (IQR 2.2-4.6 cc). Patient characteristics can be found in Table 1.

**Table 1:** Patient characteristics Gy, Gray; cc, cubic centimeters; SRS, stereotactic radiosurgery; EBRT, external beam radiotherapy; LITT, Laser-Interstitial Thermal Therapy

Patient characteristics	
Total	30
Age (years), median (25th, 75th percentiles)	60.1 (50.5, 71.5)
Gender, n(%)	
Female	17 (57%)
Male	13 (43%)
Primary, n(%)	
Breast	5 (17%)
Lung	13 (43%)
Other	12 (40%)
Whole Brain, n(%)	
Yes	8 (27%)
No	22 (73%)
Radiation dose (Gy), median (25th, 75th percentiles)	20 (18, 22)
Prescription isodose line (%), median (25th, 75th percentiles)	50 (50, 50)
Volume of metastasis treated with radiation (cc), median (25th, 75th percentiles)	1.2 (0.2, 6.6)
Time from SRS/EBRT to LITT (months), median (25th, 75th percentiles)	13.1 (9.1, 24.2)
On steroids at time of LITT, n (%)	
Yes	12 (40%)
No	17 (57%)
Unknown	1 (3%)
Volume of area with radiation necrosis at time of LITT (cc), median (25th, 75th percentiles)	2.3 (0.9, 3.7)
Treatment volume during LITT (cc), median (25th, 75th percentiles)	3.5 (2.2, 4.6)
Able to taper off steroids within 30 days of LITT, n (%)	
Yes	23 (77%)
No	7 (23%)

Clinical outcomes

Median survival of this population from the date of LITT was 2.1 years. Eighteen of 30 patients were still alive at time of last follow-up. Thirty-three percent (n=4) of patients who died (n=12) experienced neurologic death. Two patients died of progressive neurologic decline due to impaired functional status from multiple brain metastases. One patient died from progressive radiation necrosis and one patient died from leptomeningeal disease unrelated to previous brain metastases. 

**Figure 2:**
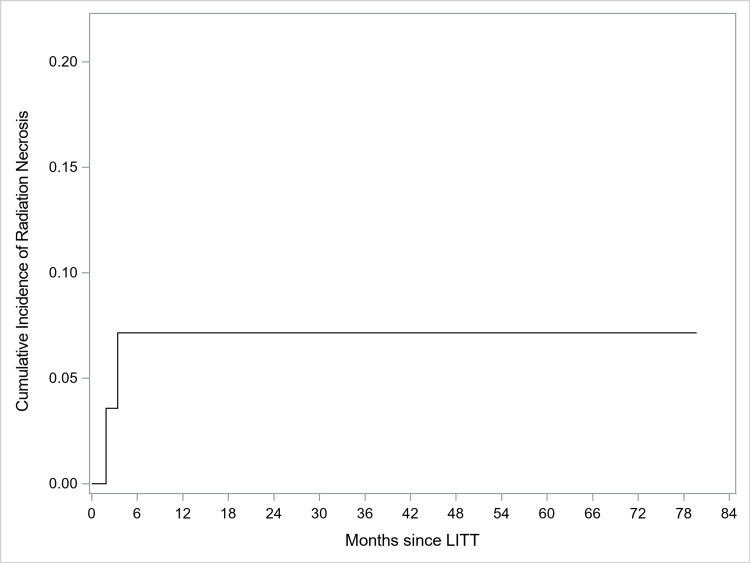
Cumulative incidence of radiation necrosis. There were two recurrences, both of which occurred less than four months after laser-interstitial thermal therapy (LITT).

Twenty-three of 30 (77%) LITT patients successfully tapered off steroids within one month of their LITT procedure. The three-, six- and nine-month freedom from RN recurrence was 95.7%, 90.9%, and 90.9%. Recurrence is defined as recurrence of symptoms after initial improvement. Two patients had recurrent RN, at 1.9 months (3.2 cc) and 3.4 months (2.9 cc) as depicted in Figure 2. They were treated successfully with bevacizumab and with craniotomy, respectively. 

Cognition and quality of life

**Figure 3:**
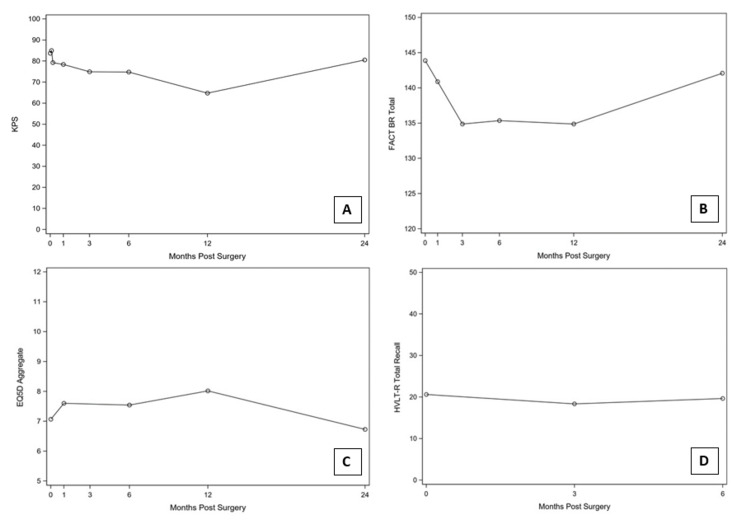
A. KPS scores from pre-procedure to 24 months post-procedure demonstrate a slight decline initially with stability over two years; 25, 15, 0, 16, 12 and 4 patients were evaluated at 0, 1, 3, 6, 12 and 24 months, respectively. B. FACT-Br total scores from pre-procedure to 24 months post-procedure demonstrate a slight decline initially with stability over two years; 22, 15, 20, 16, 10 and 2 patients were evaluated at 0, 1, 3, 6, 12, and 24 months, respectively. C. EQ-5D Aggregate scores from pre-procedure to 24 months post-procedure demonstrate stability over two years; 16, 15, 13, 9, 8 and 2 patients were evaluated at 0, 1, 3, 6, 12, and 24 months, respectively. D. HVLT-R Total Recall scores from pre-procedure to six months post-procedure demonstrate stability over time; 8, 7 and 4 patients were evaluated at 0, 3 and 6 months, respectively. FACT-Br: Functional Assessment of Cancer Therapy - Brain; EQ-5D: EuroQol-5 Dimension; HVLT: Hopkins Verbal Learning Test; KPS: Karnofsky Performance Scale

Sixteen out of 30 patients were followed with EQ-5D, 22 of 30 were followed by FACT-Br and eight of 30 were followed by HVLT instruments for measurement of cognition and quality of life. Mean Karnofsky Performance Scale (KPS) score decreased from baseline (84) to three-month follow-up (75) (p=0.03) as depicted in Figure 3A. Mean FACT-Br scores, stratified by emotional, functional, physical and social/family well being scores, were stable from baseline (17.9, 16.6, 21.4 and 22.8) to three months (18.8, 15.4, 18.4 and 23.4) (p=0.38, 0.53, 0.09 and 0.59, respectively) as depicted in Figure 3B. Mean EQ-5D Aggregate scores were stable from baseline (7.1) to one month (7.6) (p=0.25) as depicted in Figure 3C. Mean HVLT-R Total Recall scores were stable from baseline (20.6) to three months (18.4) (p=0.09) as depicted in Figure 3D. 

Complications

Only two patients (out of 30 total patients) experienced Grade 2 or higher toxicity from LITT with the highest grade of toxicity experienced being a Grade 3. Per the National Cancer Institute’s Common Toxicity Criteria Version 5.0 [18], Grade 2 toxicity indicates a moderate adverse event and Grade 3 indicates a severe and undesirable event. The single patient who experienced Grade 3 toxicity after LITT experienced an intraparenchymal hemorrhage within 24 hours of the LITT procedure. Because of the location in the left temporal lobe, the patient experienced right hemibody weakness and expressive aphasia, both of which resolved over several months. The single patient who experienced Grade II toxicity experienced subacute edema after LITT causing mild cognitive changes that were reversed with steroids.

## Discussion

There are several clinical scenarios in which patients are at higher risk of radiation necrosis, including large brain metastases [19], repeat SRS to the same lesion [18] and treatment with immunotherapy [20]. These indications have been increasing due to the trend in non-invasive management of brain metastases, the trend away from the use of whole brain radiotherapy [19] and the improved survival outcomes with immunotherapy usage amongst patients with metastatic cancer [21].

Several treatment options have emerged for radiation necrosis. Corticosteroids are the first-line treatment for patients with RN that have symptomatic edema as they can improve symptoms through reduction in edema [22]. However, patients requiring long-term corticosteroid treatment unfortunately may experience increased risk of infection, gastric ulcers, myopathies and Cushingoid changes. Bevacizumab is generally reserved for steroid-refractory RN. Bevacizumab is a monoclonal antibody against vascular endothelial growth factor (VEGF), which is involved in the signal transduction cascade leading to propagation of RN [23]. The long-term efficacy of bevacizumab remains unknown, and the Alliance for Clinical Trials in Oncology recently closed the BeST study to accrual. Bevacizumab’s risk profile includes risk of hemorrhage, thrombosis, hypertension and impaired wound healing. The combination of vitamin E and pentoxifylline has been used in cases of mild radiation necrosis as a way of improving time to imaging normalization [24]. While hyperbaric O2 has been used a treatment for radiation necrosis in the settings of radiation necrosis of benign lesions treated with SRS (e.g. arteriovenous malformation [25]), it has been considered controversial in the setting of cancer patients due to its induction of growth factors [26].

The present study demonstrates that LITT has a potentially useful role in the management of symptomatic RN. Its advantage over conventional surgical resection is its decreased invasiveness, and its ability to access deeper lesions. LITT provides the advantage of same-day biopsy so that pathological confirmation can be achieved during the same procedure. Pathological confirmation is important because the relapse rate of tumor recurrence is higher than that of RN when treated with LITT. The limitations of LITT are in treating lesions that are greater than 3 cm and those that are in eloquent cortex.

There are several limitations of the present series. The small patient numbers and variable measurements of quality of life limited the ability to statistically analyze that data. As a retrospective series, it is subject to patient selection bias. In spite of its limitations, this series represents the first series to specifically assess the response rate and durability of response of LITT in the treatment of RN caused by stereotactic radiosurgery. In spite of its small numbers, it suggests that patients tolerated the procedure well without significant worsening of quality of life metrics after treatment. 

## Conclusions

LITT is an effective and minimally invasive treatment option for patients with suspected RN. The procedure allows for same-day biopsy to differentiate between RN and recurrence. In those whose pathology demonstrates RN, LITT is a safe and durably effective treatment option.

In our population of patients with pathologically confirmed RN, there was minimal toxicity, with only two patients experiencing a Grade 2 or 3 toxicity. Only two patients had recurrences, which presented shortly after treatment and were salvageable with bevacizumab and craniotomy, respectively. Patient-reported outcomes showed no severe declines after LITT. EQ-5D, FACT-Br and HVLT questionnaires were all stable from baseline, indicating no significant change in their emotional, functional, physical and social well-being. 

LITT should be considered for definitive treatment of RN, especially in cases where patients have significant side effects from standards medical therapies such as steroids or if steroids are minimally effective. 

## References

[1] Ellis TL, Neal MT, Chan MD (2012). The role of surgery, radiosurgery and whole brain radiation therapy in the management of patients with metastatic brain tumors. Int J Surg Oncol.

[2] Weber J, Mandala M, Del Vecchio M (2017). Adjuvant nivolumab versus ipilimumab in resected stage III or IV melanoma. N Engl J Med.

[3] Kotecha R, Gondi V, Ahluwalia MS, Brastianos PK, Mehta MP (2018). Recent advances in managing brain metastasis. F1000Res.

[4] Soike MH, Hughes RT, Farris M, McTyre ER, Cramer CK, Bourland JD, Chan MD (2019). Does stereotactic radiosurgery have a role in the management of patients presenting with 4 or more brain metastases?. Neurosurgery.

[5] Johnson AG, Ruiz J, Hughes R (2015). Impact of systemic targeted agents on the clinical outcomes of patients with brain metastases. Oncotarget.

[6] Jensen CA, Chan MD, McCoy TP (2011). Cavity-directed radiosurgery as adjuvant therapy after resection of a brain metastasis. J Neurosurg.

[7] Dohm AE, Hughes R, Wheless W (2018). Surgical resection and postoperative radiosurgery versus staged radiosurgery for large brain metastases. J Neurooncol.

[8] Ayala-Peacock DN, Peiffer AM, Lucas JT (2014). A nomogram for predicting distant brain failure in patients treated with gamma knife stereotactic radiosurgery without whole brain radiotherapy. Neuro Oncol.

[9] Vellayappan B, Tan CL, Yong C (2018). Diagnosis and management of radiation necrosis in patients with brain metastases. Front Oncol.

[10] Boothe D, Young R, Yamada Y, Prager A, Chan T, Beal K (2013). Bevacizumab as a treatment for radiation necrosis of brain metastases post stereotactic radiosurgery. Neuro Oncol.

[11] Co J, De Moraes MV, Katznelson R (2020). Hyperbaric oxygen for radiation necrosis of the brain. Can J Neurol Sci.

[12] Aghajan Y, Grover I, Gorsi H, Tumblin M, Crawford JR (2019). Use of hyperbaric oxygen therapy in pediatric neuro-oncology: a single institutional experience. J Neurooncol.

[13] Missios S, Bekelis K, Barnett GH (2015). Renaissance of laser interstitial thermal ablation. Neurosurg Focus.

[14] Ahluwalia M, Barnett GH, Deng D (2018). Laser ablation after stereotactic radiosurgery: a multicenter prospective study in patients with metastatic brain tumors and radiation necrosis. J Neurosurg.

[15] Lanier CM, LeCompte MC, Glenn C (2019). Laser-interstitial thermal therapy as a novel and effective treatment in radiation necrosis following stereotactic radiosurgery to the brain. Int J Radiat Oncol Biol Phys.

[16] Shaw E, Scott C, Souhami L, Dinapoli R, Kline R, Loeffler J, Farnan N (2000). Single dose radiosurgical treatment of recurrent previously irradiated primary brain tumors and brain metastases: final report of RTOG protocol 90-05. Int J Radiat Oncol Biol Phys.

[17] McTyre ER, Johnson AG, Ruiz J (2016). Predictors of neurologic and nonneurologic death in patients with brain metastasis initially treated with upfront stereotactic radiosurgery without whole-brain radiation therapy. Neuro Oncol.

[18] (2017). Common Terminology Criteria for Adverse Events (CTCAE) Version 5.0. https://ctep.cancer.gov/protocoldevelopment/electronic_applications/docs/CTCAE_v5_Quick_Reference_5x7.pdf.

[19] Dohm A, McTyre ER, Okoukoni C (2018). Staged stereotactic radiosurgery for large brain metastases: local control and clinical outcomes of a one-two punch technique. Neurosurgery.

[20] McKay WH, McTyre ER, Okoukoni C (2017). Repeat stereotactic radiosurgery as salvage therapy for locally recurrent brain metastases previously treated with radiosurgery. J Neurosurg.

[21] Lanier CM, Hughes R, Ahmed T (2019). Immunotherapy is associated with improved survival and decreased neurologic death after SRS for brain metastases from lung and melanoma primaries. Neurooncol Pract.

[22] Kotsarini C, Griffiths PD, Wilkinson ID, Hoggard N (2010). A systematic review of the literature on the effects of dexamethasone on the brain from in vivo human-based studies: implications for physiological brain imaging of patients with intracranial tumors. Neurosurgery.

[23] Chung C, Bryant A, Brown PD (2018). Interventions for the treatment of brain radionecrosis after radiotherapy or radiosurgery. Cochrane Database Syst Rev.

[24] Williamson R, Kondziolka D, Kanaan H, Lunsford LD, Flickinger JC (2008). Adverse radiation effects after radiosurgery may benefit from oral vitamin E and pentoxifylline therapy: a pilot study. Stereotact Funct Neurosurg.

[25] Monaco EA 3rd, Niranjan A, Kano H, Flickinger JC, Kondziolka D, Lunsford LD (2013). Management of adverse radiation effects after radiosurgery for arteriovenous malformations. Prog Neurol Surg.

[26] Kang TS, Gorti GK, Quan SY, Ho M, Koch RJ (2004). Effect of hyperbaric oxygen on the growth factor profile of fibroblasts. Arch Facial Plast Surg.

